# Post-Traumatic Stress Disorder in Homeless Migrant Mothers of the Paris Region Shelters

**DOI:** 10.3390/ijerph17134908

**Published:** 2020-07-07

**Authors:** Mathilde Roze, Maria Melchior, Cécile Vuillermoz, Dalila Rezzoug, Thierry Baubet, Stéphanie Vandentorren

**Affiliations:** 1 INSERM, Sorbonne Université, Institut Pierre Louis d’Epidémiologie et de Santé Publique (IPLESP), Department of Social Epidemiology, F75012 Paris, France; mathilde.rz@gmail.com (M.R.); maria.melchior@inserm.fr (M.M.); cecile.vuillermoz@inserm.fr (C.V.); 2 CESP Inserm 1178, Université Paris 13, APHP Hôpital Avicenne, 93000 Bobigny, France; dalila.rezzoug@aphp.fr (D.R.); thierry.baubet@aphp.fr (T.B.); 3 Centre National de Ressources et Résilience, 59800 Lille, France; 4 Santé Publique France, French National Public Health Agency, F-94415 Saint-Maurice, France

**Keywords:** homeless migrants, women, PTSD, mental health

## Abstract

Migrant women are disproportionately more likely to experience traumatic events in their country of origin, during migration and after arriving in the host country. Homeless women are more likely to be exposed to multiple victimizations in childhood (emotional or physical maltreatment) and in adulthood (sexual abuse, street victimization). This study’s objective was to describe the factors associated with the likelihood of post-traumatic stress disorder (PTSD) among homeless migrant mothers in the Paris region. Face-to-face interviews were conducted by bilingual psychologists and interviewers in a representative sample of homeless families in the Paris region. PTSD was ascertained using the Mini International Neuropsychiatric Interview (MINI) (*n* = 691 mothers). We studied PTSD in mothers using weighted Poisson regression. Homeless migrant mothers had high levels of PTSD (18.9%) in the 12 months preceding the study. In multivariate analysis, PTSD was associated with departure from the country of origin because of violence (PR = 1.45 95% CI 1.03; 2.04), depression in the preceding 12 months (PR = 1.82 95% CI 1.20; 2.76), and residential instability (PR = 1.93 95% CI 1.27; 2.93). Homeless migrant mothers have high levels of traumatic events and PTSD. Improvements in screening for depression and PTSD and access to appropriate medical care are essential for this vulnerable group.

## 1. Introduction

Post-traumatic stress disorder (PTSD) is an anxiety disorder that can arise after an individual has experienced a severe traumatic event, such as the threat of injury or death (e.g., a car crash, a natural disaster, physical aggression, or rape) [1]. Symptoms include “reliving” the event with flashbacks and nightmares, avoidance with emotional numbing and feeling detached, arousal with difficulty concentrating, sleep disturbances, and outbursts of anger [2].

PTSD in homeless populations is very prevalent [3], specifically in women, with 25.7% in 123 homeless women included in the French Housing First Program [4] suffering from PTSD, versus almost 7% in women of the overall population in France in 2003 [5]. Most studies to date have explored whether homeless people have had an elevated level of exposure to traumatic events and cumulate risk factors which brought on PTSD (e.g., witnessing or being the victim of an attack, sexual assault) [6,7,8], but less is known about the role of homelessness itself as a traumatic experience that leads to exposure to factors predicting PTSD.

The number of homeless families in France has increased dramatically since 2010 due to several factors. First, the rise in poverty and the cost of living in the Paris region has increased difficulties in accessing housing, especially for the most vulnerable groups [9]. Second, the increase in the number of recent family immigrants hosted in shelters not initially intended for them has mechanically led to a higher number of homeless persons. As in many large European cities, the proportion of migrants among the homeless in Paris has increased over time (38% in 2001, 52% in 2012) [10]. The epidemiological evidence regarding the mental health of migrants remains sparse and findings are heterogeneous [11], with estimates of PTSD prevalence in adult refugees ranging from 4% [12] to 68% [13]. Migrant families are disproportionately exposed to traumatic events and comprise various profiles, such as “labor migrants”, “refugees”, and “asylum seekers”, who are particularly vulnerable to depression and PTSD [14].

First, previous studies suggest that migrants are at risk of PTSD due to a history of multiple potentially traumatic pre-migratory events [15,16]. Immigrant populations are more vulnerable to PTSD due to their exposure to traumatic experiences prior to migration, such as organized violence and political oppression. This is especially true for women [3]. Second, difficult post-migration living conditions put migrant families at risk of economic difficulties and social suffering in the host country [17]. Migrants’ families face factors associated with mental distress after migration, such as acculturative stress arising from unfamiliarity with daily tasks, discrimination, and severe post-migration difficulties, such as delays in the processing of asylum applications involving their legal status, being relegated to the margins of society, social isolation, language barriers, cultural differences, labor rights difficulties in finding stable employment and securing stable living and housing conditions, loneliness, and health difficulties [18,19].

Host countries do not take all these problems into account and in France, applying for accommodation is a complex process, particularly for immigrant families who are mobile, socially excluded, and often non-French speaking [20]. We focused on housing conditions because, first, becoming homeless can lead to trauma through the loss of stable sheltered accommodation, of connections with their family, and of social roles and routines. Second, chronic homelessness and associated stressors, such as the continuous uncertainty about where to find food and safe shelter, can erode a person’s coping mechanisms [21]. Third, homelessness might be the breaking point for those who have pre-existing behavioral health conditions or a prior history of trauma [22]. Homeless migrants’ families cumulate risk factors for developing PTSD that could impact physical [23] and other mental disorders (depression, anxiety disorders) [24,25] and also impact biological (cellular aging [26]) and social functioning [27].

To our knowledge, few studies have focused on housing conditions and homelessness and their relationship with mental health [28]. Most studies exploring PTSD in migrants have been conducted in the United States, where migrant families consist of poor young people from ethnic minorities with health issues [29]. Little is known about homeless migrant families with PTSD in Europe. Mothers with children on very low or no income constitute the majority of this population. We therefore aimed to (1) describe the prevalence of PTSD in homeless migrant mothers in the Paris region and (2) identify factors associated with PTSD, including living and housing conditions.

## 2. Methods

### 2.1. Population Study

The “*enfants et familles sans logement*” (ENFAMS; “homeless children and families”) survey [20] was conducted by the Observatoire du Samu Social from January to May 2013 to estimate the number of homeless families living in sheltered accommodation in the Paris region (approximately 12 million inhabitants) and describe their sociodemographic characteristics and health. It was based on a random sample of 801 families provided with accommodation in emergency centers, long-term rehabilitation centers, social hotels, and centers for asylum seekers. Families eligible for ENFAMS were defined as comprising at least one parent (>18 years old) with at least one child younger than 13 years, speaking one of the 17 languages of the survey, and able to provide written consent to participate.

### 2.2. Survey Design

Time–location sampling was used [30]. ENFAMS estimated that over 10,280 homeless families were living in sheltered accommodation in the Paris region, corresponding to approximately 35,000 people, including 17,660 children. The sampling process, which was detailed elsewhere [20], included three levels of random sampling: shelters (which were randomly selected among an exhaustive list of all services in the Paris region), families (which were randomly selected in each selected service; either the single parent or one of the two parents was interviewed, in 95.4% of the cases this was the mother), and one child under 13 randomly selected in each family. Families who chose not to participate in ENFAMS were characterized by younger maternal age (33 vs. 38 years), a higher proportion of men (15.3 vs. 4.6% among study participants), and having two or more children (31.7 vs. 23.1% among study participants). The reasons most frequently cited for non-participation were lack of interest (17%), lack of time (14%), or the other parent’s lack of written consent (11%).

### 2.3. Ethics

The ENFAMS survey design was approved by the French National Confidentiality Committee (CNIL, DR-2013-147)), and two ethics boards (the *Comité de Protection des Personnes* (*CPP* Ref 2012 02 06, 22/08/2012) for the greater Paris area, and the *Comité Consultatif sur le Traitement de l’Information en matière de Recherche dans le domaine de la Santé* (*CCTIRS* n_12.471, 17/09/2012)).

### 2.4. Data Collection

Survey data were collected in questionnaires during random face-to-face interviews conducted by trained interviewers and psychologists in up to 17 different languages in each shelter. For each family, the mother and one child under 13 were interviewed. Interviewing mothers was preferred when possible, given that many questions regarded very young children (e.g., breast feeding, etc.) and that they were more often present in the shelter to answer the interviewer. In our present sub-study on PTSD, we only analyzed mothers’ questionnaires (fathers only accounted for 4% of interviewed parents).

### 2.5. Variables Used

Outcome. Traumatic events were assessed using the following question: “Have you experienced a terrible, frightening, or horrible event at some point in your life that caused you to have distressing memories or nightmares, to feel isolated or distant from others, to have difficulty sleeping or concentrating, or to be excessively nervous?”. Only mothers who reported experiencing at least one traumatic event answered subsequent questions on PTSD symptoms.

PTSD (lifetime and previous 12 months) was ascertained by the PTSD module of the Mini International Neuropsychiatric Interview (MINI). The MINI is a brief structured diagnostic interview for major psychiatric disorders and generates reliable and valid psychiatric diagnoses [31,32]. Thus, the outcome of this study was “to suffer from PTSD in the 12 months preceding the study” (measured by the MINI) (yes/no).

Explanatory Variables. Factors examined as potentially associated with PTSD risk according to our analysis of the literature review included:

Sociodemographic characteristics: age; region of birth; family status (i.e., living with a partner, number of children, and their age); legal resident status; employment status; education level; typical monthly income (poverty line = 964 euros/month/UC); health insurance status (dichotomized into “full health insurance” (which included social security cover, voluntary insurance, and free healthcare for persons on low income) and “partial health insurance” (which included social security cover only or partially payable healthcare for persons on low income); proficiency in French (no difficulties to understand, to speak, to read, or to write); migratory trajectory (i.e., reason for departure from country of origin and time since family’s arrival in France).

Health: food insecurity in the preceding 12 months (assessed using the French version of the US Household Food Security Module [33,34]); self-reported health (very good, good, or fair physical health vs. poor or very poor health); serious health problem(s) disrupting daily life; anemia (ascertained by blood samples collected by study nurses); body mass index (BMI) (anthropometric measures taken by study nurses); pregnancy; female circumcision; major depression (ascertained using the Composite International Diagnostic Interview (CIDI) [35], and suicide risk (ascertained using the Mini International Neuropsychiatric Interview (MINI) [36,37]).

Living and housing conditions: previously spent at least one night on the street; type of shelter (“short-term” for social hotels or emergency shelters and “long-term” for housing facilities for asylum seekers and long-term re-habilitation centers); housing quality (i.e., number of persons per room); residential instability (i.e., less than 6 months in a current shelter); lack of social support (i.e., knowing no one living in France at the time of their arrival); help from non-governmental organizations (NGOs), friends, or family (i.e., obtaining a food voucher, luncheon voucher, food parcel, clothes, or money from these people and organizations).

### 2.6. Statistical Analysis

Our analysis was based on a sample of 691 mothers with complete PTSD data who were not born in France. Missing data were imputed (max 7.73% of missing data for health insurance status). The first step of the analysis consisted of a description of the distribution of PTSD by factors identified in our literature analysis using Chi-squared tests, using a count of those with PTSD against those with no PTSD. Second, to identify characteristics associated with PTSD in the study sample, we used a weighted Poisson regression with robust error variance [38], which provided prevalence ratios (PR) and 95% confidence intervals (95% CI). Third, all the variables significantly associated with PTSD in univariate analyses (*p* < 0.05) were included in the multivariate analysis.

All statistical analyses were performed with R software (version 3.2.0 using the “survey” weighting package and “mice” package to impute missing data).

## 3. Results

### 3.1. Study Population Characteristics

**Figure 1:**
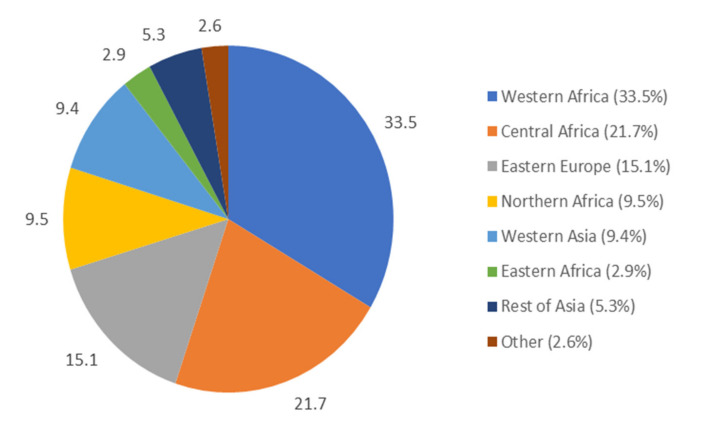
Country of origin of migrant homeless mothers in the Paris region (The “*enfants et familles sans logement*” (ENFAMS; “homeless children and families”) survey 2013, *n* = 691).

Median age at the time of the study was 31.0 years (Q1 27.0–Q3 36.0), 54.2% were born in Sub-Saharan Africa, 13.8% in North Africa, and 15.8% in Eastern Europe (Figure 1).

More than half were single mothers (53.1%), 19.2% were employed or in education, and 38.0% were proficient French speakers. Most families (98.3%) had an income below the poverty level (964 euros/month/UC), 22.2% had no health insurance, and 53.7% suffered from food insecurity. Sociodemographic characteristics for our study sample of homeless migrant mothers are shown in Table 1.

**Figure 2:**
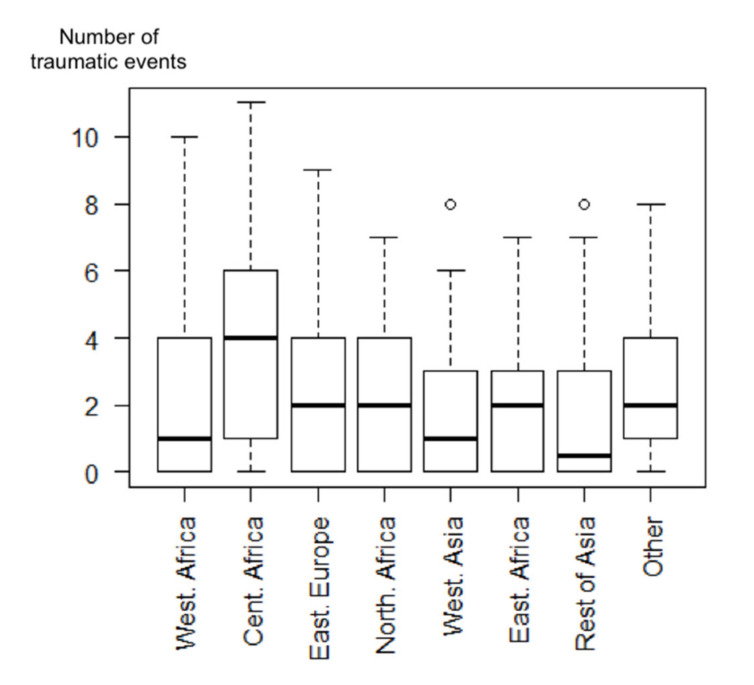
Boxplot representing the number of traumatic lifetime events according to the country of origin among migrant homeless mothers in the Paris region (ENFAMS survey 2013, *n* = 691).

Among the study population, 62.4% reported traumatic events and 16.2% had PTSD in the preceding 12 months (23.6% lifetime). Mothers born in Central Africa had a higher number of traumatic lifetime events (Figure 2), half of them having experienced at least four such events. 75% of mothers from Central Africa had experienced more than one traumatic event (first quartile), 50% more than four (median), and 25% more than six (third quartile).

The distribution of traumatic events among homeless migrant mothers with PTSD is shown in Table 2, 80.5% experienced the unexpected or sudden death of an intimate friend or family member, while 48.7% witnessed someone being injured or dying.

The majority of these women declared a good perceived health (88.3%), 25.5% were anemic, 36.6% were obese, and 7.8% were pregnant at the time of the study (Table 3). In terms of mental health, 16.9% were at risk of suicide and 27.4% had experienced depression.

Over fourteen percent (14.6%) had previously spent at least one night on the street. The median time since arrival in France was 2.7 years (Q1 1.4–Q3 5.6), 20.8% were housed in a long-term shelter, and 37.1% experienced residential instability. Over half (58.2%) did not know anyone in France when they arrived or knew someone but he/she had not helped. Finally, 27.4% had received no assistance whatsoever (from social security, friends, etc.).

### 3.2. Factors Identified as Associated with PTSD

All characteristics in Table 1 were tested for their association with PTSD. Table 3 shows only factors associated with PTSD in migrant mothers: being born outside of Europe (PR = 2.34 95% CI 1.10; 4.97); low monthly income (PR = 4.04 95% CI 1.14; 14.31); not being a proficient French speaker (PR = 0.67 95% CI 0.46; 0.98); departure from the country of origin because of violence (PR = 2.28 95% CI 1.57; 3.30); number of traumatic events (PR = 1.34 95% CI 1.27; 1.42); poor self-reported health (PR = 2.57 95% CI 1.61; 4.11); having serious health problems (PR = 1.69 95% CI 1.12; 2.55); pregnant at the time of study (PR = 2.13 95% CI 1.34; 3.38); suicide risk and depression (respectively, PR = 2.96 95% CI 2.04; 4.29 and PR = 3.83 95% CI 2.42; 6.08); having previously spent at least one night on the street (PR = 2.09 95% CI 1.40; 3.12); long-term shelter (PR = 1.54 95% CI 1.05; 2.27); residential instability (PR = 2.32 95% CI 1.54; 3.49 for mothers who had spent less than 6 months in their current shelter); and lack of social support (PR = 1.79 95% CI 1.13; 2.84).

### 3.3. Multivariate Analysis

In the multivariate analysis (Table 4), after adjusting for age, factors associated with PTSD in the study sample included departure from the country of origin because of violence (PR = 1.45 95% CI 1.03; 2.04), depression in the preceding 12 months (PR = 1.82 95% CI 1.20; 2.76), and residential instability (PR = 1.93 95% CI 1.27; 2.93).

## 4. Discussion

In our study, homeless migrant mothers reported high levels of traumatic events (62.4%) and PTSD. Specifically, 23.6% and 16.2%, respectively, met the lifetime and previous 12 months criteria for PTSD diagnosis. Departure from the country of origin because of violence (PR = 1.45) was associated with suffering from PTSD in the last 12 months. Additionally, PTSD was associated with depression in the preceding 12 months (PR = 1.82), highlighting the role of overall psychological vulnerability. Finally, post-traumatic experiences also appeared to play a role: residential instability (PR = 1.93) was associated with PTSD. Our results highlight that migrant homeless mothers accumulate stress from multiple factors, which translates into a very high, and often undetected, prevalence of PTSD.

PTSD prevalence in migrants ranges between 4 [11] and 68% [12] depending on the context and specific population under study. In a previous study [39], 42.6% and 29.7% of homeless mothers in three U.S. cities met, respectively, the lifetime and previous 12 months criteria for PTSD. Those results are consistent with ours (respectively, 23.6% and 16.2%). Previous studies on the French general population in 2001–2003 indicated that prevalence of lifetime PTSD was 3.9% and PTSD in last year was 2.2% [40].

Prior studies also found similar prevalence levels to ours with regard to traumatic events (75% to 84.15%) [13,16]. In our study, traumatic events reported by participating mothers were especially violent: 80.5% had experienced the unexpected or sudden death of an intimate friend or family member, 48.7% had witnessed someone being injured or dying, 38.4% had experienced war, 37.6% were victims of assault by a close relative, and 35.9% were victims of rape or sexual assault. To these traumatic events, we can add having spent at least one night on the street (14.6%).

Furthermore, 35.9% of our study sample were survivors of rape or sexual assault. It is important to note the growing amount of literature documenting the association between mothers’ mental health and children’s emotional difficulties [41], highlighting the importance of maternal PTSD on their offspring.

In our study, PTSD was associated with depression. Debate surrounds the question of whether it is sensible to distinguish these two diagnoses in the aftermath of trauma [42]. More specifically, some studies suggest that depression is a risk factor of PTSD, and reciprocally, that PTSD is a risk factor of depression [43], and that the two disorders represent an underlying joint vulnerability. Because our data were cross-sectional, we are not in a position to contribute to this debate. However, our results highlight a high prevalence of PTSD among migrant mothers who are psychologically vulnerable.

The post-traumatic experiences associated with PTSD in our study, such as housing accommodation and low income, are consistent with prior research. Porter and Haslam [44] found that housing accommodation and restricted economic opportunities moderated mental health outcomes, regardless of resettlement location. Several studies in the Netherlands [45] found that financial difficulties, daily stress, and immigration status [46] were associated with PTSD symptoms. In our study, living and housing conditions (residential instability), were associated with PTSD.

Social support acts as a protective factor and a buffer against the impact of traumatic events [47,48]. However vulnerable people do not usually ask for help. Our study shows that receiving assistance from an NGO, friends, or family was not associated with PTSD. However, knowing someone in France who helped the participant upon their arrival was associated with lower levels of PTSD. Other factors, such as marital status [49], French language proficiency [46], and time since arrival in France [50,51]—which may act as proxies for time since exposure to trauma—were not associated with PTSD.

Chronic PTSD could increase the risk of women’s other mental and physical health problems but also could deteriorate social functioning and impact the nearby environment and the child. Some studies demonstrated that maternal PTSD may be associated with negative child behavior outcomes (externalizing, internalizing, and emotion regulation) [52,53]. This relation could be explained because parents’ PTSD can impact the functioning of parenting (lower parenting satisfaction, less optimal parent–child relationships, and more frequent use of negative parenting practices, such as overt hostility and controlling behaviors [54]). In this context, it is necessary to develop interventions which help migrant women with PTSD, especially since some studies showed that patients with a migratory background did not benefit from psychotherapy as much as patients without a migratory background [55].

The study has several limitations. First, the ENFAMS survey was cross-sectional in nature and consequently had an exploratory approach. We did not have specific data on the history of traumatic events; therefore, it is hard to determine whether they occurred prior to the departure from the country of origin, during migration, or after arrival in France. Furthermore, the direction of the association between some of the factors studied (e.g., depression) and mental health is difficult to determine. Second, population homogeneity (most families were very poor, unemployed, etc.) may explain why some factors (e.g., food insecurity and employment status) were not associated with PTSD. Third, our participants may have differed in terms of their sensitivity to different traumatic factors according to their socio-cultural and religious backgrounds.

Despite these limitations, our study also has strengths. First, ENFAMS included a multicultural sample of homeless families rarely studied in France. In much of the existing research on PTSD, study participants are limited to individuals from the same country of origin. Our present sub-study sought to fill gaps in the mental health literature in Europe, by studying PTSD prevalence among homeless migrant mothers in the Paris area, who are diverse in terms of region of origin, legal residence status, and living and housing conditions. Second, we used validated measures for mental health (MINI and CIDI) and physical health, together with a detailed questionnaire on living and housing conditions. Third, the fact that interviews were conducted by trained interviewers and psychologists in 17 different languages provides hitherto unknown data in France.

Our results stress the role of health professionals in addressing the mental health needs of this population. Serious post-migration living difficulties, including residential instability, were related to PTSD. Serious post-migration living difficulties have a re-traumatizing effect on vulnerable individuals with limited capacity to handle resettlement stress due to their previous traumatic history [56]. Policies enhancing the social protection of immigrants in a host country would be a powerful instrument to reduce the number of traumatic events, of post-migration living difficulties, and consequent post-traumatic stress disorder. For example, permanent private accommodation is associated with better outcomes and is almost always cheaper than social hotels in France. PTSD in homeless migrant mothers may not be the inevitable consequence of acute wartime stress. Other contextual factors involved in its development and progression can be effectively managed by material and non-material support from host country governments.

## 5. Conclusions

Homeless migrant women in France have high levels of traumatic events and PTSD, in part related to their departure from their country of origin because of violence, but also to their current living and housing conditions (e.g., residential instability). Improvements in the monitoring of mental health (depression and PTSD) and access to appropriate medical care for this vulnerable population could limit the impact of PTSD on their health and on that of their children.

## Figures and Tables

**Table 1:** Sociodemographic characteristics of homeless migrant mothers in the Paris region (ENFAMS survey 2013, *n* = 691).

			Whole Population	With PTSD (*n* = 120)		Without PTSD (*n* = 571)		
		Weighted Proportion	IC	Weighted Proportion	IC	Weighted Proportion	IC	*p*
Sociodemographic Characteristics								
Mother’s age	(17–27) y.o.	23.4	(19.5; 27.4)	19.7	(12.6; 26.7)	24.2	(19.5; 28.8)	0.333
	(27–57) y.o.	76.6	(72.6; 80.5)	80.3	(73.3; 87.4)	75.8	(71.2; 80.5)	
Region of birth	Europe	15.9	(12.3; 19.5)	7.5	(2.0; 13.0)	17.5	(13.4; 21.6)	0.019
	Outside Europe	84.1	(80.5; 87.7)	92.5	(87.0; 98.0)	82.5	(78.4; 86.6)	
Family status	Two-parent family	46.9	(42; 51.8)	38.3	(28.7; 47.9)	48.6	(43.4; 53.8)	0.052
	Single mother	53.1	(48.2–58)	61.7	(52.1; 71.3)	51.4	(46.2; 56.6)	
Legal residence status	Already acquired legal status	14.3	(10.7; 17.8)	15.5	(5.2; 25.9)	14.0	(10.2; 17.8)	0.939
	Undocumented	48.3	(43.5; 53.1)	48.0	(36.6; 59.5)	48.4	(43.3; 53.4)	
	Asylum seeker	12.1	(9.7; 14.5)	13.0	(7.8; 18.1)	11.9	(9.2; 14.6)	
	Temporary residence permit	25.3	(21.2; 29.4)	23.5	(15.7; 31.2)	25.7	(20.9; 30.5)	
Employment status	Employed or in education	19.2	(15.3; 23.1)	13.2	(4.9; 21.5)	20.4	(16.0; 24.8)	0.180
	Neither employed nor in education	80.8	(76.9; 84.7)	86.8	(78.5; 95.1)	79.6	(75.2; 84.0)	
Educational level	<high school	50.5	(46.1–54.9)	40.0	(28.1; 51.8)	52.4	(47.6; 57.3)	0.066
	≥high school	49.5	(45.1–53.9)	60.0	(48.2; 71.9)	47.6	(42.7; 52.4)	
Usual monthly income	Above poverty line	1.7	(0.5; 3)	0.4	(0; 0.9)	2.0	(0.5; 3.5)	0.013
	Below poverty line	98.3	(97; 99.5)	99.6	(99.1; 100)	98.0	(96.5; 99.5)	
Health insurance	Complete	66.7	(62.1; 71.2)	69.6	(57.7; 81.5)	66.2	(61.5; 70.8)	0.090
	Partial	11.1	(8.4; 13.9)	4.2	(1.4; 7.0)	12.3	(9.1; 15.6)	
	None	22.2	(18.3; 26)	26.2	(14.3; 38.1)	21.5	(17.6; 25.4)	
Food insecurity	No	46.3	(41.3; 51.2)	42.7	(31.7; 53.7)	47.0	(41.5; 52.4)	0.492
	Yes	53.7	(48.8–58.7)	57.3	(46.3; 68.3)	53.0	(47.6; 58.5)	
French Language proficiency	Yes	38	(32.3; 43.7)	47.7	(36.2; 59.1)	36.1	(30.3; 42.0)	0.042
	No	62	(56.3; 67.7)	52.3	(40.9; 63.8)	63.9	(58.0; 69.7)	
Departure from country of origin because of violence	No	70	(66; 74)	50.3	(39.2; 61.4)	73.8	(70.0; 77.5)	<0.001
	Yes	30	(26; 34)	49.7	(38.6; 60.8)	26.2	(22.5; 30.0)	
Number of traumatic events	(min; Q1; med; mean; Q3; max)			(0; 3; 5; 4.7; 6; 11)		(0; 0; 1; 2.0; 4; 10)		0.008
Time of traumatic event(s)	Traumatic event before arriving in France	38.4	(33.7; 43)	75.4	(64.8; 86.0)	31.0	(26.6; 35.3)	<0.001
	Traumatic event after arriving in France	22.6	(18.3; 26.8)	24.6	(14.0; 35.2)	22.1	(17.7; 26.6)	
	No traumatic event	39.1	(33.9; 44.3)	0.0	(0; 0)	46.9	(41.2; 52.6)	

**Table 2:** Traumatic lifetime events in homeless migrant mothers who suffered from Post-traumatic stress disorder (PTSD) in the last 12 months, Paris region (ENFAMS survey 2013, *n* = 120).

Traumatic Lifetime Events	Women with PTSD (*n* = 149) %
Unexpected or sudden death of an intimate friend or a member of family	80.5
Witnessing someone get injured or dying	48.7
Experienced a wartime event	38.4
Victim of assault by a close relative	37.6
Accident or life-threatening illness	36.1
Victim of rape or sexual assault	35.9
Victim of torture or kidnapping	33.6
Discovery of a corpse	29.0
Victim of burglary or armed robbery	25.6
Earthquake, landslide, hurricane	18.9
Other disasters including fires, flooding	13.2
Victim of physical assault in the course of work	8.8
Illness of an intimate friend or family member	4.7
Responsible for injury or death of another person	3.2
Exposure to radiation or other dangerous substances	1.4

**Table 3:** Health characteristics and living conditions of homeless migrant mothers in the Paris region (ENFAMS survey 2013, *n* = 691).

Health Characteristics and Living Conditions		Whole Population		With PTSD (*n* = 120)		Without PTSD (*n* = 571)		
		Weighted Proportion	IC	Weighted Proportion	IC	Weighted Proportion	IC	*p*
Health Characteristics								
Perceived current general state of health	Very good, good or fair global health	88.3	(85.4; 91.2)	74.7	(63.3; 86.1)	91,0	(88.4; 93.5)	<0.001
	Poor or very poor global health	11.7	(8.8; 14.6)	25.3	(13.9; 36.7)	9,0	(6.5; 11.6)	
Serious health problem(s)	No	71.3	(66.8; 75.8)	59.4	(47.9; 70.9)	73,6	(69.1; 78.1)	0.012
	Yes	28.7	(24.2; 33.2)	40.6	(29.1; 52.1)	26,4	(21.9; 30.9)	
Pregnant (at time of survey)	No	92.2	(90; 94.3)	84.7	(77.2; 92.2)	93,6	(91.6; 95.6)	0.003
	Yes	7.8	(5.7; 10)	15.3	(7.8; 22.8)	6,4	(4.4; 8.4)	
Female circumcision	No	79.3	(75.4; 83.1)	81.6	(73.2; 90.1)	78,8	(74.5; 83.1)	0.584
	Yes	20.7	(16.9; 24.6)	18.4	(9.9; 26.8)	21,2	(16.9; 25.5)	
Suicide risk	No	83.1	(79.7; 86.5)	62.5	(52.1; 72.9)	87,1	(83.7; 90.5)	<0.001
	Yes	16.9	(13.5; 20.3)	37.5	(27.1; 47.9)	12,9	(9.5; 16.3)	
Depression in the previous 12 months	No	72.6	(68.7; 76.5)	40.3	(28.1; 52.6)	78,8	(74.7; 83.0)	<0.001
	Yes	27.4	(23.5; 31.3)	59.7	(47.4; 71.9)	21,2	(17.0; 25.3)	
Living Conditions								
Previously spent at least one night on the street	No	85.4	(82.6; 88.3)	73.4	(64.4; 82.4)	87,8	(84.8; 90.7)	<0.001
	Yes	14.6	(11.7; 17.4)	26.6	(17.6; 35.6)	12,2	(9.3; 15.2)	
Time since arrival in France	>1 year	85.3	(82.5; 88)	84.8	(77.9; 91.6)	85,4	(82.5; 88.3)	0.864
	≤1 year	14.7	(12; 17.5)	15.2	(8.4; 22.1)	14,6	(11.7; 17.5)	
Type of shelter	Short-term shelter	79.2	(75.5; 83)	71.2	(61.7; 80.7)	80,8	(77.0; 84.5)	0.031
	Long-term shelter	20.8	(17; 24.5)	28.8	(19.3; 38.3)	19,2	(15.5; 23)	
Number of persons per room	≤2 pers per room	42.1	(36.5; 47.7)	44.4	(33.8; 55.0)	41,6	(35.8; 47.5)	0.613
	>2 pers per room	57.9	(52.3; 63.5)	55.6	(45.0; 66.2)	58,4	(52.5; 64.2)	
Residential instability	No	62.9	(58.2; 67.6)	42.3	(31.6; 52.9)	66,9	(61.7; 72.2)	<0.001
	Yes	37.1	(32.4; 41.8)	57.7	(47.1; 68.4)	33,1	(27.8; 38.3)	
Already knew someone who lived in France, and this person helped	Yes, and he/she was helpful	41.8	(37.2; 46.4)	28.6	(18.2; 39.0)	44,3	(39.4; 49.3)	0.013
	No, I did not know anyone or yes, but he/she was not helpful	58.2	(53.6; 62.8)	71.4	(61.0; 81.8)	55,7	(50.7; 60.6)	
Help from association, friends or family	Yes	72.6	(68.3; 76.9)	69.1	(58.6; 79.5)	73,2	(68.6; 77.9)	0.454
	No	27.4	(23.1; 31.7)	30.9	(20.5; 41.4)	26,8	(22.1; 31.4)	

**Table 4:** Characteristics associated with PTSD in the last 12 months in homeless migrant mothers in the Paris region (ENFAMS survey 2013, *n* = 691, univariate and multivariate Poisson regression analyses).

	Characteristics		Univariate Analysis			Multivariate Analysis	
		Weighted Proportion	PTSD Prevalence	PR	IC	PR	IC
Socio-Demographic Characteristics							
Mother’s age	(17–27) yo	23.4	13.6	-			
	(27–57) yo	76.6	17.0	1.25	(0.79; 1.98)		
Country of birth	Europe	15.9	7.6	ref			
	Outside Europe	84.1	17.8	2.34	(1.10; 4.97)		
Typical monthly income	Above poverty line	1.7	4.1	-			
	Below poverty line	98.3	16.4	4.04	(1.14; 14.31)		
Proficiency in French language	Yes	38.0	20.3	-			
	No	62.0	13.7	0.67	(0.46; 0.98)		
Departure from country of origin because of violence	No	69.8	11.7	-		ref	
	Yes	30.2	26.6	2.28	(1.57; 3.30)	1.45	(1.03; 2.04)
Number of traumatic events	(min; Q1; med; mean; Q3; max)	(0; 0; 1; 2.2; 4; 11)		1.34	(1.27; 1.42)		
Time of traumatic event (TE)	TE before arrival in France	39.0	31.3	-			
	TE after arrival in France	23.2	17.2	0,55	(0.34;0.88)		
Health Characteristics							
Perceived current general state of health	Very good, good or fair global health	88.4	13.7	-			
	Poor or very poor global health	11.6	35.2	2.57	(1.61; 4.11)		
Serious health problem(s)	No	71.2	13..5	-			
	Yes	28.8	22.8	1,69	(1.12; 2.55)		
Pregnant at time of study	No	92.2	14.9	-			
	Yes	7.8	31.7	2,13	(1.34; 3.38]		
Suicide risk	No	83.1	12.2	-			
	Yes	16.9	36.0	2,96	(2.04; 4.29)		
Depression in the previous 12 months	No	72.7	9.1	-			
	Yes	27.3	35.0	3.83	(2.42; 6.08)	1.82	(1.20; 2.76)
Living Conditions							
Previously spent at least one night on the street	No	85.3	13.9	-			
	Yes	14.7	29.1	2.09	(1.40; 3.12)		
Type of shelter	Short-term shelter	79.2	14.5	-			
	Long-term shelter	20.8	22.4	1.54	(1.05; 2.27)		
Residential instability	No	62.9	10.9	-		ref	
	Yes	37.1	25.2	2.32	(1.54; 3.49)	1.93	(1.27; 2.93)
Already knew someone who lived in France, and he/she helped	Yes, and he/she was helpful	41.9	11.1	-			
	No, I did not know anyone or yes, but he/she was not helpful	58.1	19.8	1.79	(1.13; 2.84)		
